# A Small Surge in Incidence of SARS-CoV-2 Omicron Variant in the “Dynamic Zero” Period

**DOI:** 10.1155/2023/5262117

**Published:** 2023-03-13

**Authors:** Xiaona Li, Ruili Li, Qiguo Lian, Yang Wang, Wenkui Gu, Qinghe Meng

**Affiliations:** ^1^Beichen District Center for Disease Control and Prevention, Tianjin, China; ^2^Children Health and Development Department, Capital Institute of Paediatrics, Beijing, China; ^3^NHC Key Lab. of Reproduction Regulation (Shanghai Institute for Biomedical and Pharmaceutical Technologies), Fudan University, Shanghai, China

## Abstract

To describe the epidemiological characteristics and transmission dynamics of SARS-CoV-2 Omicron variant during “Dynamic Zero” period, we analyzed data on the 108 laboratory-confirmed SARS-CoV-2 cases during 14 to 30 May 2022 in Beichen district, Tianjin, China. We collected information on demographic characteristics, exposure history, and illness timelines of the 108 cases. We described characteristics of the patients and estimated the key epidemiological parameters, including serial interval and the time-dependent reproduction number of the Omicron variant, Rt. Among the 108 laboratory-confirmed patients, the median age was 38 years old, and 50.9% were females. Obvious symptoms were observed among 67.6% (73/108) of all cases, and major clinical manifestations included fever, sore throat, and cough, which occurred in 31.5%, 26.9%, and 19.4% of the 108 cases, respectively. The mean and standard deviation of the SI were estimated as 2.89 and 0.95 days, the Rt varied from 1.24 to 0.27 for a 7-day timelapse. The low reproduction number and the Omicron outbreak being suppressed within a short time marked the effectiveness of the implemented public health measures, such as nucleic acid screening, social distancing, masking, vaccination, medical treatment of patients, and isolation of close contacts. These measures play an important role in fulfilling the goal of controlling the spread of the disease.

## 1. Introduction

The severe acute-respiratory syndrome coronavirus 2 (SARS-CoV-2) has evolved rapidly into new variants throughout the pandemic. The Omicron variant has been identified globally in numerous countries [1]. An increased growth rate of Omicron cases is attributable to increased transmissibility, a shorter generation time, or both of these factors [2, 3]. In January 2022, the initial local case of SARS-CoV-2 variant Omicron occurred in Tianjin, China. Then, the variant became dominant in China quickly [4]. We implemented “Dynamic Zero” policy in China ever since the initial cases of SARS-CoV-2 occurred in Wuhan, China. On 15 May 2022, nucleic acid testing of SARS-CoV-2 was performed on all staff in Beichen district, Tianjin, China. A small surge in incidence of SARS-CoV-2 Omicron variant (BA.2) was observed. Control measures such as timely isolating the patients and close contacts, disinfection of the patient's environment, nucleic acid testing of people in high-risk area and medium-risk area, and lockdown of the epidemic community were implemented immediately. Various studies have modeled the transmissibility of SARS-CoV-2 variant but most were carried out in the pandemic environment [5–7]. In this research, we focused on quantifying the transmissibility at the time when we implemented the “Dynamic Zero” policy. We calculated the time-varying reproduction number (Rt) using the serial interval (SI), which can provide policy makers real-time estimates of disease transmission potential to guide decisions on containment measures.

## 2. Materials and Methods

Epidemiological investigations throughout the epidemic often provide valuable data. When the case was confirmed as positive to SARS-CoV-2, a joint-field epidemiology team comprising members from municipal CDC and Beichen district CDC would open a detailed field investigation on demography information, epidemiological histories, timelines of key events, and close contacts. In this study, we collected dates of illness onset for 21 infector-infectee pairs from case epidemiological reports and calculated the serial interval from these data. The serial interval distribution (SI) is defined as the time duration between the symptom onset in the primary case (infector) and symptom onset in the secondary case (infectee) and indicates the interval between the two infected patients [8]. The date of illness onset was defined as the date on which a symptom relevant to SARS-CoV-2 infection appeared. The date for confirmed cases without symptoms was depicted as their confirmation date. In this study, the SI was fitted by a gamma distribution with infector/infectee pairs. The effective reproduction number (Rt) was defined as the average number of cases directly generated by one case over a predefined time window. This value was important in assessing the extent of epidemic transmission, predicting the future trends, and designing the control measures. In general, an epidemic will increase as long as Rt is greater than 1, and control measures aim to reduce the reproduction number to less than 1.

Analyses of the serial interval and time-varying reproduction number were performed with the use of R software version 4.2.0 (R Foundation for Statistical Computing). We used the “EpiEstim” package to estimate the effective reproduction number of SARS-CoV-2 Omicron variant. In the “EpiEstim” package, estimates of reproduction number over predefined time windows can be obtained within the Bayesian framework [9].

## 3. Results

**Figure 1:**
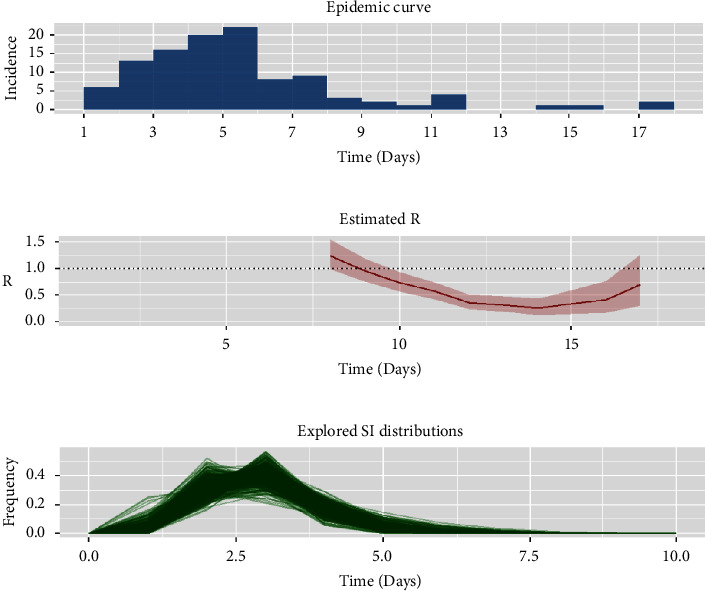
Transmissibility assessment of SARS-CoV-2 Omicron variant in Beichen district, Tianjin, China. (a) Epidemic curve of reported confirmed cases (*n* = 108). (b) The time-dependent reproduction number (95% CI) of Omicron variant. (Using the Markov Chain Monte Carlo method and for 7-day timelapse on moving average data). (c) The distribution of serial interval of Omicron variant.

From 14 to 30 May 2022, a total of 108 laboratory-confirmed cases were monitored in Beichen district, Tianjin, China. The last case was reported on 30 May. The peak number of single-day cases was on the 5th day (18 May) with the number of cases being 22 (Figure 1(a)). The median age of all confirmed cases was 38 years (ranges from 1 year to 83 years), and 50.9% were females. Among all cases, 32.4% (35/108) patients were asymptomatic, and most of the 108 SARS-CoV-2 omicron cases were with mild symptoms, and no cases were with severe disease or death. 95.4% (103/108) cases were vaccinated, 7.4% (8/108) cases got one dose of vaccine, 48.1% (52/108) cases got two doses of vaccine, and 39.8% (43/108) cases got a booster shot. Our analysis showed that there were 25 (23.1%) cases detected by community screening, 63 (58.3%) cases detected by testing of the close contact, and 20 (18.6%) cases detected through high- and medium-risk area screening. The detailed data are shown in Table 1.

We obtained 21 infector/infectee pairs from case epidemiological reports; the Gamma distribution provided the best fit for the SI of the outbreak. The estimated serial interval distribution had a mean (±SD) of 2.89 ± 0.95 days (Figure 1(c)). Using the serial interval distribution discussed above, we estimated that the time-varying reproduction number was 1.24 to 0.27 (Figure 1(b)). It was notable that the effective reproduction number was below 1 one week later ever since the public health preventive measures were implemented, which indicated that the outbreak was effectively controlled.

## 4. Discussion

In this study, we showed the epidemiological characteristics and the transmission capacity of SARS-CoV-2 Omicron variant in Beichen district, Tianjin, in the “Dynamic Zero” period. There were no severe cases appeared probably due to vaccination; though the variant has some capacity to evade immunity, modest levels of neutralizing antibodies might protect people from severe forms of COVID-19 [10]. It took us seven days to make the effective reproduction number fall below 1 and 16 days to achieve the goal of no new case reported, which showed that we had achieved significant progress in preventing the spread of the epidemic. Reports from different sources confirm the presence of asymptomatic carriers; asymptomatic carriers can also cause transmission and should be considered as a source of infection in epidemiological investigations [11, 12]. Besides, it has been verified by various studies that presymptomatic transmissions are occurring [13, 14], so containment via rapid case-finding and isolation of all those infected under the test-trace-isolation strategy are likely to be very challenging. Of the 108 cases, there were 63 (58.3%) cases detected by testing of the close contact and 20 (18.6%) cases detected through high- and medium-risk area screening, both of which were in quarantined population. In absence of timely preventive and control measures, those could cause a mass epidemic in the community.

Various studies have focused on assessing the transmissibility of SARS-CoV-2 Omicron variant. To compare with the different estimates of other studies, we conducted an analysis using the classical epidemic model extensively used by researchers. Our estimate of the serial interval as 2.89 ± 0.95 days was similar to a previous study in Korea [15], which also belonged to small-sample research (the case number was 80) but shorter than another study [16]; the discrepancy is likely due to the small sample size in our study, as well as the different levels of immunity in affected population, including both of vaccine-induced immunity and natural immunity acquired from previous infection. The shorter serial interval of SARS-CoV-2 Omicron variant indicates that the infection can lead to rapid cycles of transmission from one generation of cases to the next, which also implies that contact tracing and isolating must compete against the rapid replacement of case generations; as by the time contacts are traced, they may have already become infectious themselves and generated secondary cases.

The time-varying reproduction numbers are good indicators of the speed of disease spread and can be used for understanding the impacts of control strategies in real time. If Rt < 1, it suggests that the epidemic is in decline and may be regarded as under control at time t (vice versa, if Rt > 1) [17]. To eradicate an epidemic, Rt needs to remain below 1. The effective reproduction number of Beichen district was a declining curve in the intense period of this outbreak, and Rt was reduced below 1 since 22 May (7 days after the outbreak). Our analysis showed that the Rt varied from 1.24 to 0.27, apparently lower than the other research reports [1, 6, 18], which attributed to the timely implemented public health measures against the transmission of the Omicron variant and low incidence in the period when we carried out “Dynamic Zero” policy. The reproduction number may change over time due to other factors such as seasonal variations in the parameters governing disease spread [19–21], which we did not take into account when comparing reproduction numbers with other studies.

This study has some limitations. First, the estimation was calculated upon small sample size, the value of SI and Rt could be biased, and the transmissibility might not be generalized elsewhere. Second, the effect of vaccine on progression of disease was not assessed; a case-controlled study in which different groups of participants matched in terms of age and health conditions was expected to be carried out in future to evaluate the effect of vaccine. Finally, the clinical characteristics were not compared with other studies.

## 5. Conclusions

A lesson from the epidemic is that a rapid response to the infectious disease is essential. With the joint efforts of the government and the public, the outbreak can be controlled quickly and effectively, which can also mitigate the impact of the outbreak on society and socioeconomic conditions.

## Figures and Tables

**Table 1:** Demographic and epidemiological characteristics of cases with SARS-CoV-2 Omicron variant in Beichen district, Tianjin, China.

Characteristics	No. (%) of patients
Sample size	108
Median age	38
Symptom profile	
Symptomatic	73 (67.6)
Asymptomatic	35 (32.4)
Age group	
<15 years	10 (9.3)
15–29	16 (14.8)
30–44	37 (34.3)
45–59	39 (36.1)
60+	6 (5.6)
Gender	
Male	53 (49.1)
Female	55 (50.9)
SARS-CoV-2 vaccination status	
Unvaccinated	5 (4.6)
One dose	8 (7.4)
Two doses	52 (48.1)
Booster injection	43 (39.8)
Symptoms	
Fever	34 (31.5)
Sore throat	29 (26.9)
Cough/sputum	21 (19.4)
Myalgia	8 (7.4)
Running nose	6 (5.6)
Headache	4 (3.7)
Chill	2 (1.9)
Diarrhea	2 (1.9)
Case detection method	
High- and medium-risk area screening	20 (18.6)
Testing of close contact	63 (58.3)
Community screening	25 (23.1)

## Data Availability

The data used to support the findings of this study are available at https://wsjk.tj.gov.cn/.
